# Auditory findings and electrophysiologics in individuals with G/BBB syndrome

**DOI:** 10.1590/S1808-86942011000600014

**Published:** 2015-10-19

**Authors:** Tatiana Vialôgo Cassab, Sthella Zanchetta, Célia Maria Giacheti, Neivo Luiz Zorzetto, Antonio Richieri-Costa

**Affiliations:** 1 Master's degree, doctoral graduate student in the science of rehabilitation of craniofacial anomalies, HRAC, Sao Paulo University, Bauru, Sao Paulo, Brazil; 2 Doctoral degree, professor in the speech therapy course, Ribeirao Preto Medical School, FMRP/USP; 3 Adjunct professor of speech therapy diagnosis, Speech Therapy Department, Paulista State University, UNESP, Marilia, Sao Paulo, Brazil; 4 Full professor of speech and auditory organ anatomy and physiology, Speech Therapy Department, Paulista State University, UNESP, Marilia, Sao Paulo, Brazil; 5 Livre-Docente, medical geneticist in the Craniofacial Anomaly Rehabilitation Hospital, Sao Paulo University, USP, Bauru, SP

**Keywords:** evoked potentials, auditory, brainstem, hearing, hypertelorism, hypospadias

## Abstract

**Abstract:**

The G/BBB syndrome is a rare condition characterized by hypertelorism, cleft lip and palate, and hypospadias. No studies were found on the hearing of individuals with this syndrome.

**Aim:**

To investigate the auditory function in patients with G/BBB syndrome, such as the occurrence of hearing loss, and central and peripheral auditory nerve conduction.

**Methods:**

Fourteen male patients aged 7-34 years with the G/BBB syndrome were assessed by otoscopy, audiometry, tympanometry and evoked auditory brainstem response (ABR). Model: A retrospective clinical series study.

**Results:**

Audiometric thresholds were normal in 12 (66.7%) of the sample and altered in two (33.3%), one with conductive and one with sensorineural loss. ABR resuts were: all patients had normal absolute wave I latencies; absolute wave III and V latencies were increased in two and six patients, respectively; interpeak latencies I-III, IV and V interpeak latencies were increased in four, three and eight patients respectively.

**Conclusion:**

Hearing loss can occur in the G/BBB syndrome. There is evidence of central auditory pathway involvement in the brainstem. Imaging studies are needed to clarify the clinical findings.

## INTRODUCTION

The G/BBB syndrome was first described in 196^9^,^1^; it was subsequently divided into two other syndromes. The G syndrome was described in a family whose name started with G and in which four people had this condition; the BBB syndrome was described in three families whose names started with B – in this case there were eight people with the disease. Later, it was concluded that the G and BBB syndromes were part of the same condition. The findings in this disease consist of body midline defects, mostly in the craniofacial skeleton and the genital tubercule. The main features for a clinical diagnosis are the presence of telecanthus and hypospadia in males, and telecanthus and hypospadia in male relatives of these patients. The prevalence of this disease is unclear; reported cases show that there are 37 men for every 31 women affected^2^. No data was found about the prevalence in different races.

The genetic transmission of this disease remains unknown, but it should be autosomal dominant or X-linked due to a specific mutation in the MID1 gene. Recent genetic molecular studies have shown that it is a heterogeneous condition located in the chromosome 22 (region q 11.^2^,^3^, ^4^, ^5^, ^6^, ^7^, ^8^, ^9^, ^10^, ^11^, ^12^) and in chromosome X (region p 2^2^,^9^,^10^).

There are several papers in the literature that associate craniofacial anomalies and hearing loss. A specific study^13^ reported that 58 of 71 syndromes and congenital anomalies associated with deafness had a craniofacial anomaly of some kind. Congenital craniofacial anomalies may be associated with hearing loss, especially if cleft palate is part of the clinical picture. The G/BBB syndrome is a rare condition among all craniofacial syndromes that have been reported; it consists of three main anomalies: hypertelorism, cleft lip and palate, and hypospadia (other anomalies may be present). Cleft lip and palate is a congenital condition that stands out among those with complex changes, especially conditions that cause otologic problems and hearing loss. These changes in patients with cleft palate are related with poor function of the auditory tube resulting from insufficient palatine velum tensor muscle, which in turn causes functional obstruction of the tuba and negative pressure in the middle ear, thereby giving rise to otitis media and/or hearing loss^14^, ^15^, ^16^, ^17^, ^18^.

A high rate of hearing loss in this population together with frequent episodes of otitis media may alter auditory feedback and deprive these patients of verbal experiences and full social life. This type of sensory deprivation may lead to incomplete speech and language development and impaired learning^18^.

Because of the number of patients with a genetic and clinical diagnosis of the G/BBB syndrome registered at the Craniofacial Anomaly Rehabilitation Hospital (USP) and the lack of studies on hearing in these patients, a systematic investigation of this group is needed.

The G/BBB syndrome is a congenital malformation characterized by an altered midline – hypertelorism, hypospadia, cleft lip and palate, and laryngeal anomalies. Other findings in these patients are: imperforated anus, impaired development, and malformed hearts^19^. Included among the main findings in the G/ BBB syndrome are central nervous system anomalies: agenesis or hypoplasia of the corpus callosum, the Dandy-Walker anomaly, widening of the cisterna magna, a widened fourth ventricle, widening of the superior cerebellar cistern, atrophic vermis, dilated ventricles, cerebral atrophy, and asymmetric hemispheres^19^, ^20^, ^21^, ^22^.

We did not find studies on peripheral or central hearing in patients with the G/BBB syndrome. There is little information about the behavioral manifestation of these patients. Thus, the purpose of this study was to investigate peripheral and central hearing in patients with a diagnosis of the G/BBB syndrome to verify the occurrence of hearing loss, and to study peripheral and central brainstem nervous conduction.

## MATERIAL AND METHOD

The institutional review board approved this study, according to the requirements of Resolutions 196/96 and 251/97. The approval protocol number was 376/2006-UEP-CEP.

The sample comprised 14 male patients aged from 7 to 34 years with a clinical and genetic diagnosis of the G/BBB syndrome. All patients had an operated cleft palate. The test battery consisted of pure tone and voice audiometry, tympanometry, and BAEP.

Ipsilateral recordings were made with 1 to 4 kHz 0.1 ms clicks (the main range was between 2, 3, and 4 kHz) using 24 clicks per second. The analysis time was 10 ms; if needed, a filter was used to exclude frequencies below 300 Hz. Waves were recorded by adding 1,000 alternated polarity stimuli; the intensity was 80 dB, and three recording were made at each intensity. Stimuli were presented by means of overthe-ear phones (*HORTMANN Neuro-Otometrie, Beyerdynamic* DT48).

## RESULTS

There was bilateral hearing loss in 14.3% (2/14) of the sample. Patient 2 had moderate hearing loss (mean 43.7 dBHL bilaterally). Patient 9 had mild hearing loss (mean 22.5 dBHL in the right ear and 21.25 dBHL in the left ear).

Tympanometry was done in 13 of 14 patients because one patient had ventilation tubes bilaterally. Thus, in 26 tested ears, (19 - 73%) had type A curves, meaning that the middle ear was intact. An Ad curve was present in three ears (11.5%). A type B curve was present in two ears (7.7%). A type As curve was present in two ears (7.7%) (Table 1).

**Table 1:** Distribution of AV pure tones, means, and interpretation of results per ear and per patient.

Patient	ATL	250	500	1	2	3	4	6	8	X 0.5,1,2,4	Interpretation per ear	Final interpretation
1	RE	10	10	5	10	5	5	10	15	7.5	normal	Normal
	LE	15	15	5	10	5	5	20	20	8.75	normal	
2	RE	55	50	45	40	50	40	55	75	43.75	altered	Bilateral hearing loss
	LE	55	50	45	40	50	40	55	75	43.75	altered	
3	RE	10	10	15	10	10	10	15	15	11.25	normal	Normal
	LE	10	10	10	15	15	15	10	15	12.5	normal	
4	RE	10	15	10	10	5	5	10	10	10	normal	Normal
	LE	15	15	5	15	20	15	10	5	12.5	normal	
5	RE	-	15	10	5	10	10	-	-	10	normal	Normal
	LE	-	5	10	10	5	10	-	-	10	normal	
6	RE	10	10	10	10	10	5	5	10	8.75	normal	Normal
	LE	10	10	5	5	10	10	10	10	7.5	normal	
7	RE	5	5	5	5	0	0	5	5	3.75	normal	Normal
	LE	10	5	5	5	5	5	0	5	5	normal	
8	RE	10	10	10	10	15	15	10	10	11.25	normal	Normal
	LE	10	10	15	15	10	10	10	15	12.5	normal	
9	RE	10	10	10	5	40	65	55	60	22.5	altered	Bilateral hearing loss
	LE	10	5	10	10	40	60	15	10	21.5	altered	
10	RE	20	15	15	10	15	20	25	25	15	normal	Normal
	LE	20	15	15	20	10	10	20	10	15	normal	
11	RE	10	15	10	10	15	10	10	15	11.25	normal	Normal
	LE	10	10	10	10	15	10	10	10	10	normal	
12	RE	5	5	5	5	5	5	10	10	5	normal	Normal
	LE	5	5	5	10	10	5	10	5	6.25	normal	
13	RE	20	20	15	15	15	15	20	20	16.25	normal	Normal
	LE	20	20	20	15	10	10	20	20	16.25	normal	
14	RE	-	20	15	20	20	15	-	-	17	normal	Normal
	LE	-	20	15	15	15	15	-	-	17.5	normal	

As we concluded, 14.3% (2/14) had bilateral hearing loss. Patient 2 had moderate hearing loss (mean 43.7 dBHL bilaterally). Patient 9 had mild hearing loss (mean 22.5 dBHL in the right ear and 21.25 dBHL in the left ear) (Table 2).

**Table 2:** Occurrence of hearing loss in G/BBB patients.

Patient	ATL		250	500	1	2	3	4	6	8	Tympanometric curve
P2	VA	OD	50	50	45	40	50	40	55	75	Type B
		OE	55	50	45	40	50	40	55	75	Type B
	VO	OD	-	0	0	0	5	5	-	-	-
		OE	-	0	0	0	5	0	-	-	-
P9	VA	OD	10	10	10	5	40	60	55	60	Type A
		OE	10	10	10	10	40	60	15	10	Type Ad
	VO	OD	-	-	-	-	30	60	-	-	-
		OE	-	-	-	-	30	60	-	-	-

Tympanometry was done in 13 of 14 patients because one patient had ventilation tubes bilaterally.

Thus, in 26 tested ears, 19 (73%) had type A curves, meaning that the middle ear was intact. An Ad curve was present in three ears (11.5%). A type B curve was present in two ears (7.7%). A type As curve was present in two ears (7.7%).

Two patients with peripheral hearing loss were excluded from the 14-patient sample. Thus, the study population comprised 12 subjects.

Table 3 presents the mean values of the LA and LIP parameters in patients.

**Table 3:** Distribution of BAEP minimum, maximum, mean values, and standard deviation of waves I, III, and V latencies and interpeaks I-III, III-V, and I-V in right and left ears of G/BBB syndrome patients.

Side	Variables	Minimum	Maximum	Mean	SD
Right ear	I	1,7	1,9	1,81	0,082
	III	3,9	4,2	4,03	0,093
	V	5,7	6,4	5,95	0,183
	I-III	2,0	2,4	2,20	0,121
	III-V	1,7	2,3	1,92	0,168
	I-V	3,8	4,7	4,13	0,212
Left ear	I	1,8	2,0	1,89	0,085
	III	3,5	4,2	3,97	0,166
	V	5,6	6,3	5,99	0,172
	I-III	1,8	2,3	2,09	0,106
	III-V	1,9	2,2	2,01	0,119
	I-V	3,9	4,3	4,10	0,122
V i.a.		0	0,4	0,12	0,114

The 12 patients had waves I, III, and V bilaterally with good morphology. Four patients (33% of the sample) had normal latency values. Eight patients (66.7%) had higher values and were considered as having altered results; this was bilateral in six patients and unilateral in two. No patient had a wave V interaural difference over 0.4 ms.

Table 4 presents the mean values of the LA and LIP BAEP parameters in the entire series.

**Table 4:** BAEP results in patients with the G/BBB syndrome (latencies in ms).

Subjects	Ear	I	III	V	I-III	III-V	I-V	V i.a.
1	RE	1,7	4	5,7	2,3	1,7	4	0,1
	LE	1,8	3,5	5,6	1,8	2,1	3,9	
3	RE	1,9	4,2	6	2,3	1,8	4,1	0,1
	LE	2	4	6,1	2	2,1	4,1	
4	RE	1,8	4	5,9	2,2	1,9	4,1	0,1
	LE	1,8	3,9	5,8	2,1	1,9	4	
5	RE	1,9	3,9	6	2	2,1	4,1	0
	LE	1,8	3,9	6	2,1	2,1	4,2	
6	RE	1,8	4,1	6	2,2	2	4,2	0,3
	LE	2	4,1	6,3	2,1	2,2	4,3	
7	RE	1,9	3,9	5,7	2	1,8	3,8	0,3
	LE	2	4,1	6	2,1	1,9	4	
8	RE	1,7	3,9	5,9	2,2	2	4,2	0
	LE	1,8	3,8	5,9	2	2,1	4,1	
9	RE	1,7	4,1	6,4	2,4	2,3	4,7	0,3
	LE	2	4,1	6,1	2,1	1,9	4	
11	RE	1,8	4,1	6,1	2,3	2	4,3	0,1
	LE	1,9	4,2	6,2	2,3	2	4,3	
12	RE	1,9	4,1	5,8	2,2	1,7	3,9	0,1
	LE	1,8	4	5,9	2,2	1,9	4,1	
13	RE	1,9	4,1	6	2,2	1,9	4,1	0,1
	LE	1,9	4,1	6,1	2,2	2	4,2	
14	RE	1,9	4	6	2,1	2	4,1	0,1
	LE	2	4	6,1	2	2,1	4,1	

Absolute wave I latencies were within normal limits in all patients.

Absolute wave III latency values were increased unilaterally in two patients; absolute wave V latencies were increased in four patients (one bilaterally and three unilaterally).

Interpeak I-III latency values were increased in four patients (one bilaterally and three unilaterally). Interpeak III-V values were increased unilaterally in two patients. Interpeak I-V values were increased bilaterally in two patients and unilaterally in four patients out of six.

## DISCUSSION

No direct comparison with the literature we reviewed was possible, as no studies on hearing in the G/BBB syndrome were found. Our data show that four of 14 patients reported suspected hypoacusis and a history of repeated events of otitis; two cases had abnormal audiometries. Otitis media in patients with cleft palate has been well documented^14^,^15^; it may cause auditory complaints, even though serous otitis media may present few symptoms. A few authors^23^ have noted that most unoperated cleft palate patients with hearing loss reported no auditory complaints. Although most patients in this study presented cleft palates, the majority had no auditory complaints; only two had peripheral hearing loss.

Pure tone audiometry was done in all subjects; these results were analyzed quantitatively and qualitatively.

Quantitatively, two of 14 subjects had bilateral hearing loss; one was sensorineural (mild grade) and one was conduction (moderate grade). Both were compatible with the tympanometry – normal middle ear in the former and abnormal middle ear in the later – and the audiologic interview data were also concordant. Similarly, sensorineural hearing loss in the abovementioned patient may be explained by abnormal conduction and frequent recurrences^23^, ^24^, ^25^.

The cleft lip and palate population may be considered as vulnerable to hearing loss because of poor auditory tube function due to abnormally inserted elevator and tensor palatine velum muscles^4^,^8^. The study patients had normal hearing, which agreed with the findings of tympanometry.

Subjects in the study sample were aged 7 to 34 years; all had undergone palatoplasty. These factors may have contributed to a low rate of conduction abnormalities; many published papers have reported that hearing improves significantly after this procedure because the anatomy and function of palatine velum muscles is restablished^26^, ^27^, ^28^, ^29^, ^30^. Additionally, in both the cleft palatine and normal populations, middle ear abnormalities are more common in infancy because the auditory tube is more horizontal at this age^31^.

Normal values obtained in biological gauging^32^ for the genetics clinic of the Craniofacial Anomaly Rehabilitation Hospital *(Hospital de Reabilitação de Anomalias Craniofaciais* or HRAC) were used in the analysis of BAEP results.

There were no patients without the main analyzed waves; however, most of the sample (66.7%) had increased absolute and interpeak latencies.

Absolute wave III latencies (generated in the cochlear nucleus and the olivary complex), wave V latencies (generated in the lateral lemniscus and the inferior olivary complex), and interpeaks I-III, III-V, and I-V were increased in eight of 12 subjects in this study compared to normal limits.

Thus, the results suggest that patients with the G/BBB syndrome present delayed conduction of the acoustic stimulus along auditory pathways. Altered neural conduction of sound stimuli in the presence of normal audiometric thresholds have been described^33^. These authors found that normal hearing subjects had increased waves III and V latencies and interpeaks I-III and IV in BAEP.

Conduction abnormalities cause absolute latencies to increased in BAEP^30^,^34^,^35^; this explains why BAEP was not done in two patients with peripheral auditory abnormalities.

Central nervous system involvement may explain altered BAEPs in this study; structural central nervous system abnormalities have been reported in the literature as being one of the main symptoms of the G/BBB syndrome^19^, ^20^, ^21^, ^22^. However, we were unable to confirm central nervous system involvement in our subjects because of technical hurdles that made it difficult to carry out image tests; this is one of the limitations of this study. This issue may be clarified in future studies correlating audiologic and image tests.

Craniofacial anomalies associated with genetic syndromes are considered risk factors for impaired hearing^36^. In the present study, cleft lip and palate in all subjects may be considered a study bias; the institution in which the study was undertaken is a reference center for craniofacial abnormalities and cleft lip and palate. This study underlines the importance of investigating the auditory system in patients with the G/BBB syndrome, as normally functioning peripheral and central auditory pathways have an important role in speech development and social interaction.

## CONCLUSION

Our findings indicate that patients with the G/ BBB syndrome may present peripheral, conductive, and sensorineural hearing loss. However, there were no data stating that these losses are due to the syndrome or if they are associated with cleft palate.

There is evidence of central auditory pathway involvement in the brainstem, even though central nervous system abnormalities in the G/BBB syndrome are not related directly with auditory pathways.

Studies on the audiologic profile of this population and imaging are needed to clarify the clinical findings.
